# UK guidelines for the management of bone sarcomas

**DOI:** 10.1186/s13569-016-0047-1

**Published:** 2016-05-04

**Authors:** Craig Gerrand, Nick Athanasou, Bernadette Brennan, Robert Grimer, Ian Judson, Bruce Morland, David Peake, Beatrice Seddon, Jeremy Whelan, On behalf of the British Sarcoma Group

**Affiliations:** Newcastle upon Tyne Hospitals NHS Foundation Trust, Freeman Hospital, Newcastle upon Tyne, NE7 7DN UK; Nuffield Orthopaedic Centre, Oxford, OX3 7LD UK; Royal Manchester Children’s Hospital, Manchester, M13 9WL UK; Royal Orthopaedic Hospital, Birmingham, B31 2AP UK; The Royal Marsden, Sutton, SM2 5PT UK; Birmingham Children’s Hospital, Birmingham, B4 6NH UK; Queen Elizabeth Hospital, Birmingham, B15 2TH UK; University College Hospital, London, NW1 2PG UK

## Abstract

This document is an update of the British Sarcoma Group guidelines published in 2010. The aim is to provide a reference standard for the clinical care of patients in the UK with bone sarcomas. Recent recommendations by the European Society of Medical Oncology, The National Comprehensive Cancer Network and The National Institute for Health and Care Excellence have been incorporated, and the literature since 2010 reviewed. The standards represent a consensus amongst British Sarcoma Group members in 2015. It is acknowledged that these guidelines will need further updates as care evolves. The key recommendations are that bone pain or a palpable mass should always lead to further investigation and that patients with clinico-radiological findings suggestive of a primary bone tumour at any site in the skeleton should be referred to a specialist centre and managed by a fully accredited bone sarcoma multidisciplinary team. Treatment recommendations are provided for the major tumour types and for localised, metastatic and recurrent disease. Follow up schedules are suggested.

## Background

### Rationale and objective of guidelines

Bone sarcomas are uncommon malignancies and it was recognised more than 30 years ago that their management should be centralized. Following various NHS reforms, the diagnosis and surgical treatment of primary bone sarcomas is now commissioned by the NHS England Highly Specialized Commissioning Group [1] in five centres in England. However, other treatments such as chemotherapy and radiotherapy may be delegated to other centres. Arrangements in Scotland and Northern Ireland differ. The surgical treatment of patients in Wales takes place in specialist centres in England, with other modalities of treatment delivered within Wales.

This reference document aims to improve the quality of care for patients with bone tumours by identifying and informing key management decisions and is an update of the 2010 British Sarcoma Group (BSG) guidelines [2].

### Methods

In developing these guidelines, the following were consulted: The National Comprehensive Cancer Network (NCCN) Clinical Practice Guidelines [3]; ESMO/European Network Working Group, Clinical Practice Guidelines for Bone Sarcomas [4]; National Institute for Health and Care Excellence (NICE) Quality Standard [QS78] Sarcoma [5] and Suspected cancer: recognition and referral guideline [NG12] [6] and the published literature from 2010 to 2015. The authors considered the applicability to UK practice and reached consensus on the content. The document was then circulated widely within the British Sarcoma Group for comment and approval.

### Scope of guidelines

The guidelines apply to all primary bone sarcomas (and giant cell tumours of bone) arising in any skeletal location. These guidelines consider clinical effectiveness, and include treatments to which a specialist bone sarcoma multidisciplinary team (MDT) in the UK should have access. While representing a broad consensus in 2015, these guidelines will require updating as treatment evolves. Haemopoietic tumours of bone, rehabilitation, prosthetic services and palliative care are not included.

## Classification of bone sarcomas

Primary malignant bone tumours comprise 0.2 % of all cancers diagnosed in England and have an annual incidence of around 7.9 per million [7]. On average, 380 people were diagnosed with primary bone sarcomas each year in England between 1985 and 2009. Therefore, a General Practitioner (GP) is unlikely to see a patient with a bone sarcoma in a working lifetime. Delays in diagnosis are common. Reducing delays would almost certainly lead to improved survival outcomes and less extensive surgery [8].

Despite their rarity, primary malignant bone tumours comprise approximately 5 % of all childhood cancers in European Countries [7, 9] and include two major cancers of children and young adults: osteosarcoma and Ewing sarcoma [7]. In children under 5 years of age, a destructive bone lesion is more likely to be metastatic neuroblastoma or eosinophilic granuloma [4, 10]. Chondrosarcoma is more common in middle aged and elderly people [7].

In adults, especially those over 40 years of age, metastatic carcinomas (usually from lung, breast, thyroid, kidney or prostate) and haemopoietic malignancies (e.g. plasma cell tumour or lymphoma) in bone considerably outnumber primary bone tumours. At any age the possibility of a benign lesion or infection must be considered [11]. If there is diagnostic uncertainty, it should be assumed the patient has a primary bone sarcoma until proven otherwise [12].

There has been no significant improvement in 5-year overall survival rates for patients with bone sarcomas over the past 25–30 years, with rates static at between 53 and 55 % [7, 13].

A classification of bone sarcomas adapted from the World Health Organization (WHO) classification of primary bone tumours is shown in Table 1 [14].

**Table 1:** Classification of malignant primary bone tumours (adapted from WHO classification [14])

Chondrogenic tumours	(1) Atypical cartilaginous tumour/chondrosarcoma (grade I) (2) Chondrosarcoma (grades II/III) (3) Dedifferentiated chondrosarcoma (4) Mesenchymal chondrosarcoma (5) Clear cell chondrosarcoma
Osteogenic tumours	(1) Low-grade central osteosarcoma (2) Conventional (high-grade) osteosarcoma (chondroblastic fibroblastic osteoblastic) (3) Telangiectatic osteosarcoma (4) Small cell osteosarcoma (5) Secondary osteosarcoma (6) Parosteal osteosarcoma (7) Periosteal osteosarcoma (8) High-grade surface osteosarcoma
Notochordal tumours	Chordoma
Vascular tumours	(1) Epithelioid haemangioendothelioma (2) Angiosarcoma
Other malignant mesenchymal tumours	Fibrosarcoma, Leiomyosarcoma, Liposarcoma etc.
Miscellaneous tumours	(1) Ewing sarcoma (2) Adamantinoma (3) Undifferentiated high-grade pleomorphic sarcoma of bone

Although some inherited and acquired factors are associated with the development of primary bone tumours, a cause cannot be identified in the majority of patients [15, 16].

The 5-year relative survival for patients diagnosed in England in 1985–2004 is considerably lower than that reported within the US Surveillance, Epidemiology and End Results (SEER) programme (66 %) [17]. This may reflect the fact that patients in the SEER programme are younger (30 % of NCIN patients were >65 years, compared with 21 % of SEER patients: 22 % of NCIN patients were <19 years, compared with 29 % of SEER patients). Furthermore, the SEER programme does not specify which morphological sub-types are included. Further investigation is required [7].

### Osteosarcoma

Osteosarcoma is the second most frequent primary cancer of bone and accounts for over 10 % of all solid cancers in adolescents (age 15–19). Another peak in incidence occurs in the seventh and eighth decades of life [7, 13]. It is slightly more common in males (male to female ratio 1.4:1.0) [7]. Survival rates are significantly higher in younger patients (5-year survival, <40 years 53 % vs. >40 years 22 %; p < 0.0001) [7, 13].

Osteosarcoma usually arises in the metaphysis of an extremity long bone, most commonly around the knee [18, 19]. Some tumours (predominantly in adults) arise in the axial skeleton, pelvis or craniofacial bones. Risk factors for osteosarcoma include previous radiation therapy, Paget’s disease of bone and germline abnormalities such as Li-Fraumeni syndrome, Werner syndrome, Rothmund -Thomson syndrome and familial retinoblastoma [20, 21]. The temporal association of osteosarcoma with the pubertal growth spurt and the location in the metaphysis of long bones suggest an association with rapid bone growth.

### Ewing sarcoma

Ewing sarcoma is the second most common primary malignant bone tumour in children and adolescents, but is also seen in adults. The median age at diagnosis is around 15 years and in the UK there is a male preponderance of 1.5:1 [7]. It is less common in people of Chinese or Black African origin. Identification of chromosomal translocations specific to Ewing sarcoma e.g. (t11;22) have provided a useful diagnostic criterion. In recent years undifferentiated bone sarcomas with morphological features of Ewing sarcoma but with uncharacteristic translocations e.g. CIC-DUX have been described. Although clinical information is limited these appear to respond less well than Ewing sarcoma to chemotherapy and may have an unfavourable prognosis [22].

The most frequent anatomical sites of involvement of Ewing sarcoma are the long bones, pelvis, ribs and vertebral column. All forms of Ewing Sarcoma are high grade [23, 24].

### Chondrosarcoma

Chondrosarcoma most commonly presents between 30 and 60 years of age [7]. The ageing UK population means that chondrosarcoma has become the most common bone sarcoma, ahead of osteosarcoma [25]. Differentiating between an atypical enchondroma and a low grade chondrosarcoma can be extremely difficult and has led to these tumours being categorised together in the WHO classification as atypical cartilaginous tumour/chondrosarcoma grade I. It is considered to be a tumour of intermediate malignancy, most often behaving in a locally aggressive fashion and rarely metastasising. Care must be taken not to overtreat benign tumours or undertreat malignant ones [26].

Most chondrosarcomas are located in long bones but they also arise in flat bones (e.g. pelvis, rib and scapula). Chondrosarcomas arising in pre-existing benign lesions such as osteochondromas and enchondromas are known as secondary peripheral chondrosarcomas and secondary central chondrosarcomas respectively. The risk of developing chondosarcoma in solitary osteochondromas and enchondromas is uncertain, but is increased when there are multiple lesions or when lesions are located in the axial skeleton, particularly the pelvis [27].

The majority of primary chondrosarcomas are low- rather than high-grade [28] and are of the conventional subtype. Rare subtypes include mesenchymal chondrosarcoma and clear cell chondrosarcoma. In rare circumstances, conventional chondrosarcomas “dedifferentiate” into very high-grade tumours (so-called de-differentiated chondrosarcoma) [29–31] with a poor prognosis.

### Undifferentiated pleomorphic sarcoma of bone

Undifferentiated pleomorphic sarcoma (UPS) of bone is a relatively recent term for sarcomas that do not exhibit a specific line or pattern of differentiation (previously termed malignant fibrous histiocytoma of bone) [32, 33]. UPS of bone is typically high-grade with metastatic rates of at least 50 % [33]. Treatment usually involves neoadjuvant therapy followed by wide excision. Its chemosensitivity and survival rate are similar to osteosarcoma [34]. Occasionally, an undifferentiated pleomorphic sarcoma is found to be a dedifferentiated chondrosarcoma or osteosarcoma after resection.

### Chordoma

Chordomas develop from persistent notochordal elements, and originate from the sacrum (50 %), skull base (30 %), and mobile spine (20 %). Extraskeletal tumours are very rare. Chordoma is a locally invasive, typically low-grade tumour but infrequently (around 5 %) highly malignant dedifferentiated cases occur [35]. Metastases develop in 30–40 % of patients, typically late in the disease trajectory and usually after local recurrence. Metastases can occur in lung, liver, bone, subcutis, lymph nodes and other sites.

### Adamantinoma

Adamantinoma is a rare, low-grade malignant neoplasm that arises in the tibia, fibula or both bones, although it has rarely been reported in other bones [36]. Adamantinoma accounts for 0.3–1 % of all malignant bone tumours and occurs mostly in young to middle-aged adults (20–40 years of age), with a male-to female ratio of 1.3:1. The tibial shaft (medial or distal) is most commonly affected. The tumour has lytic and sometimes destructive areas which can lead to fracture [37]. Recurrence is late (can be >20 years) but frequent (about 30 %) after incomplete excision. The rate of metastasis is 10 % to 20 %, usually to lung [36].

### Giant cell tumour of bone

Giant cell tumours of bone are generally considered benign but locally aggressive tumours; there is a low risk of metastasis, particularly after local recurrence [38]. Giant cell tumours rarely appear before skeletal maturity and most often affect patients between 20 and 30 years of age [39]. Tumours characteristically occur at the end of a long bone in a juxtaarticular location. Histologically tumours contain a proliferation of mononuclear stromal cells amongst which are scattered numerous multinucleated giant cells that have been identified as osteoclasts recruited by the RANK-ligand expressing stromal cells. Tumours cause local destruction of bone and may be associated with a soft tissue mass or pathological fracture [40].

### Other malignant mesenchymal tumours

Very rarely malignant mesenchymal tumours that more commonly arise in soft tissues can present as a primary (often spindle cell) sarcoma of bone. These include spindle cell malignancies such as leiomyosarcoma and fibrosarcoma. In general, spindle cell sarcomas are thought to represent between 2 and 5 % of primary bone malignancies. Spindle cell sarcomas arise in a similar age group to chondrosarcoma but the skeletal distribution is similar to osteosarcoma. There is a high incidence of fracture at presentation. Associations with pre-existing conditions (e.g. Paget’s disease or bone infarct) or previous irradiation have been reported [41].

## Presentation and referral

The most common symptom of a primary malignant bone tumour is pain, which may gradually increase in intensity [42]. Bone pain at night should always be considered a ‘red flag’ symptom requiring further investigation. Pain levels may vary and a bone swelling or soft tissue mass may develop later. Even high-grade tumours do not usually cause systemic symptoms; when present these may indicate metastatic disease [42]. The average duration of symptoms is 3 months, although 6 months or longer is not uncommon [8, 43, 44].

A plain x-ray is the first investigation of choice. The presence of any of the following X-ray features is suggestive, but not diagnostic, of a primary bone tumour and should be investigated further, usually following urgent referral to a bone sarcoma MDT:

- Bone destruction
- New bone formation
- Periosteal swelling
- Soft tissue swelling

Additionally, it must be remembered that a ‘normal’ x-ray does not rule out bone sarcoma; persistent bone pain/night pain should still require urgent MRI scan/referral to a sarcoma centre. Hip and knee pain in children is often attributed to sporting injury with early ‘normal’ looking x-rays.

In all patients a full clinical history should be taken (including duration, intensity and diurnal variation of pain, prior benign or malignant tumours, family history and previous radiotherapy) and examination performed (with specific attention to the size, consistency, mobility, and location in relation to bone of any mass and palpation of regional and local lymph nodes), considering the most likely diagnosis for a patient of a given age. Recent injury does not rule out a primary bone tumour and should not prevent further examination.

In patients under 40 years of age, investigations prior to referral should include X-ray of the affected bone (in two planes) and simple blood tests [full blood count (FBC), erythrocyte sedimentation rate (ESR), biochemical profile including alkaline phosphatase (ALP)]. Further urgent imaging of the local site with magnetic resonance imaging (MRI) or computed tomography (CT) is usually required, prior to or after referral [8].

In patients over 40 years of age more extensive investigation before referral is appropriate (if it can be done quickly) as the most likely diagnosis is of metastatic carcinoma in bone. Appropriate investigations include CT of chest, abdomen and pelvis, isotope bone scan, and myeloma screen. If the bone lesion is solitary the patient should be referred to a reference centre to exclude a primary malignant bone tumour.

All patients with a possible diagnosis of a primary bone tumour should be referred urgently under the 2 week wait pathway to a fully accredited bone sarcoma MDT [4, 45, 46]. This core principle is embedded in the NICE ‘Improving Outcomes for People with Sarcomas’ guidance [47], ‘Children and Young People with Cancer’ guidance [48] and the NICE Quality Standard QS78 Sarcoma [5].

Referral before biopsy is essential to ensure optimal diagnosis and management [49, 50] since poorly planned or executed biopsies can compromise future treatment [42].

Networks should ensure GPs are aware of and comply with the urgent referral criteria in the NICE Suspected cancer: recognition and referral guideline NG12 [6] and that GPs and hospital doctors are aware of the local diagnostic pathways for patients with suspected primary bone tumours. There are also referral guidelines specific to Scotland which can be found at healthcare improvements Scotland [51].

### UK reference centres

Royal National Orthopaedic Hospital Stanmore, London

 Phone 020 8909 5112

 Fax 020 8909 5709

 www.londonsarcoma.org

 www.lsesn.nhs.uk

 rno-tr.LondonSarcomaService@nhs.net

Royal Orthopaedic Hospital Northfield, Birmingham

 Phone 0121 685 4150

 Fax 0121 685 4146

Nuffield Orthopaedic Centre Oxford

 Phone 01865 738061

 Fax 01865738037

North of England Bone and Soft Tissue Tumour Service,

 Freeman Hospital

 Newcastle upon Tyne

 Phone 0191 233 6161 or 0191 213 7708

 Fax 0191 233 1328

 www.newcastlesarcoma.org.uk

Greater Manchester and Oswestry Sarcoma Service,

 Robert Jones and Agnes Hunt Hospital

 Oswestry

 Phone 0845 838 3429

 Fax 0845 838 3428

Scotland: The Scottish Sarcoma Network,

 Glasgow Royal Infirmary

 Glasgow G4 0SF

 Phone 0141 232 1034 or 07951 273920

 www.ssn.scot.nhs.uk

In Scotland sarcomas are managed under the umbrella of the Scottish Sarcoma Network, which includes centres in Edinburgh, Glasgow and Aberdeen with a shared MDT. Links to the three national networks can be found at the West of Scotland Cancer Network (WoSCAN) [52].

Northern Ireland Sarcoma Network

 Musgrave Park Hospital

 Stockmans Lane

 Belfast

 BT9 7JB

 Tel (028) 95046964

 Mob 07885238652

 Fax (028) 90637423

### Key recommendations

- The most common symptom of a primary bone tumour is pain which may gradually increase or vary in intensity. Bone pain at night should always be considered a ‘red flag’ symptom requiring further investigation.
- The presence of pain or a palpable mass arising from any bone requires further investigation. A plain X-ray is the first investigation of choice.
- The presence of radiological features including bone destruction, new bone formation, periosteal swelling and/or soft tissue swelling are suggestive, but not diagnostic, of a bone tumour and require further investigation.
- Networks should ensure that GPs are aware of and comply with the urgent referral criteria in the NICE ‘Suspected cancer: recognition and referral guidlines’ and ‘Cancer referral guidelines for Scotland’ and that GPs and hospital doctors are aware of the diagnostic pathways for patients with suspected primary bone tumours.
- All patients with a provisional histological and/or radiological diagnosis of bone sarcoma should have their diagnosis reviewed by a specialist sarcoma pathologist and/or radiologist, both of whom should be part of a bone sarcoma MDT.

## Investigation

### Imaging

All patients should have X-rays in two planes at presentation.

Further local site imaging should be with MRI [42], including the whole anatomical compartment, the involved bone and adjacent joints [53]. CT is helpful if there is diagnostic uncertainty or MRI is contraindicated, and may better visualise areas of microcalcification, periosteal bone formation and cortical destruction. CT is routinely used in addition to MRI for pelvic tumours. Dynamic contrast enhanced MRI may identify high-grade areas within a chondrosarcoma, and therefore guide biopsy.

Staging investigations for patients with confirmed primary malignant bone tumours should include chest radiography and/or CT. CT is the technique of choice for imaging the chest, pelvis and mandible [53–55]. If indeterminate nodules are detected in the lungs, an interval scan may be indicated. All suspicious chest CTs should be reported by a radiologist experienced in bone sarcoma or sent to an MDT for review.

Whole body bone scintigraphy will detect lesions elsewhere in the skeleton [24]. Whole body MRI and positron emission tomography (PET) may be considered for staging and treatment response evaluation [54, 56–58]. A recent retrospective study of 91 patients with Ewing sarcoma [57], concluded that F-18-deoxy-d-glucose positron emission tomography (^18^FDG-PET) may be sufficient for initial screening of osseous metastases and also identified all patients with bone marrow metastases.

During chemotherapy clinical assessment (pain and clinical measurement) and imaging of the local site and lungs by MRI, chest X-ray and CT may be helpful to evaluate response to chemotherapy [53, 59].

### Staging systems

Two staging systems are in widespread use, the Enneking [60] and the TNM system (American Joint Committee on Cancer–AJCC/International Union against cancer–UICC) [61].

The Enneking system is based on histological grade (I = low and II = high grade) and extent in relation to the anatomical compartments of the limb (a = intracompartmental, b = extracompartmental). If the bone cortex is intact and there is no soft tissue mass, the tumour is considered intracompartmental. Stage III tumours have metastases, but can be high or low grade. The TNM (AJCC/UICC) system is based on tumour grade, size and the presence of metastases (Table 2).

**Table 2:** AJCC/UICC Staging [61]

Stage	Grade	Size (cm)	Metastases
1°	Low grade	≤8	None
1b	Low grade	>8	None
2°	High grade	≤8	None
2b	High grade	>8	None
3	Any grade	Any	Skip metastases
4	Any grade	Any	Distant metastases at diagnosis

### Laboratory tests

There are no specific laboratory tests for the diagnosis of bone sarcoma. However, the following are of prognostic value: erythrocyte sedimentation rate (ESR), alkaline phosphatase (ALP) and lactate dehydrogenase (LDH) [62, 63].

### Other baseline assessments

Around 10 % of Ewing sarcomas metastasise to bone marrow, and therefore bone marrow biopsy should be routinely performed as a staging investigation [42].

Chemotherapy treatment can result in renal, cardiac and auditory dysfunction [64]. Pretreatment evaluation should therefore include baseline renal function testing (e.g. urea, creatinine, glomerular filtration rate) and assessment of cardiac function (e.g. echocardiogram, MUGA [multi-gated acquisition scan]). An audiogram is recommended for patients due to receive cisplatin.

Sperm storage is recommended for male patients of reproductive age. For female patients, a fertility physician may be consulted to discuss options for fertility preservation.

### Biopsy

Biopsy is the definitive diagnostic test. Biopsy of a suspected primary malignant bone tumour should be carried out at a specialist sarcoma reference centre by, or in consultation with, the surgical team who will perform definitive tumour resection [47]. This improves access to modern molecular diagnostic techniques and ensures the biopsy track can be excised at the time of definitive surgery. Inappropriate biopsy can compromise limb salvage or even cure. The principles of biopsy [49] are:

- Biopsy should only be done after local imaging of the affected bone to allow planning of the approach and most representative area to biopsy.
- There should be minimal contamination of normal tissues.
- In many situations, core needle biopsy will be adequate, often guided by ultrasound, X-ray or CT.
- Samples should always be taken for microbiological assessment as well as histology and cytogenetic/molecular genetic studies.
- Where possible, samples should be snap frozen for storage in a tumour bank for future research studies with patient consent.
- Samples must be interpreted by an experienced bone tumour pathologist.
- The pathology request form should ensure sufficient detail to make a diagnosis, including anatomical site, patient age and the radiological differential diagnosis.

CT-guided biopsies [65, 66] are most appropriate for deeper locations (e.g. pelvis) or to target a particular area of concern within the tumour (e.g. a possibly dedifferentiated area in a chondrosarcoma). Frozen sections can help to confirm that lesional tissue has been obtained, but they should not be relied upon for a definitive diagnosis and may use up a significant volume of potentially diagnostic material. Biopsy tracks should be clearly marked with a small incision or tattoo to ensure they are excised at the definitive procedure.

Biopsy of other indeterminate lesions should always be considered if management might change as a result (e.g. entry into a trial or a decision to amputate).

Laminectomy or decompression for spinal tumours should be avoided at diagnosis unless necessary to relieve spinal cord compression, and after consultation with a member of the bone sarcoma MDT.

### Pathology

Pathologists reporting biopsies and/or resections of bone sarcomas should be accredited bone tumour pathologists and members of a bone sarcoma MDT.

Reports should comply with Royal College of Pathologists guidance [67].

The biopsy report should include a description of the specimen, the microscopic findings and the histological diagnosis.

The pathology report relating to the definitive resection specimen should include a gross description recording the location and size (measured in three dimensions in mm) of the tumour. It should note the extent of local tumour spread and involvement of specific anatomical compartments. Resection margins should be reported as clear or involved by tumour. The distance (in mm) of infiltrating tumour from the nearest resection margin and the nature of tissue at this margin should be specified. The histological features of the tumour and results of relevant further investigations (e.g. immunohistochemistry or molecular genetics) should be recorded. The tumour type (and subtype) should be recorded in keeping with the latest WHO criteria [14]. The tumour type should be coded using the systematized nomenclature of medicine—clinical terms (SNOMED-CT) codes [68].

### Molecular genetics and pathology

Tissue banks are essential for diagnostic and translational research in cancer; therefore, informed consent for tumour banking, analysis and research should be sought according to local practice wherever possible. In specialist centres, storage of fresh frozen tissue should be undertaken in every case where consent has been given.

Although most Ewing sarcomas can be recognised morphologically and by immunohistochemical identification of the surface glycoprotein CD99, molecular genetic confirmation of a Ewing sarcoma translocation is recommended, particularly if the clinicopathological presentation is unusual or the histological diagnosis is doubtful. A reference laboratory for Ewing sarcoma diagnosis should have both interphase fluorescence in situ hybridisation (FISH) and reverse transcription—polymerase chain reaction (RT-PCR) technology available [69] and should participate in an external quality assurance programme.

### Confirmation of diagnosis

To confirm the diagnosis and minimise the risk of diagnostic and management errors, all patients with suspected bone tumours should be discussed by a bone sarcoma MDT with a surgeon, radiologist, pathologist and oncologist who have access to the relevant information and biopsy material [47].

### Key recommendations

- Patients with suspected primary bone tumours should have access to timely and appropriate imaging.
- The definitive diagnostic test is a biopsy, which should be carried out at or in consultation with the team in a reference centre.
- All patients should have tissue stored for subsequent investigation with appropriate consent, including frozen tissue, when possible.
- Both the diagnostic and resection specimens should be examined by an accredited bone tumour pathologist who is part of a bone sarcoma MDT. The pathology report should comply with the Royal College of Pathologists guidance.
- In every case the diagnosis must be confirmed by reference to clinical findings, laboratory investigation and radiological imaging at a bone sarcoma MDT.
- Patients with a confirmed diagnosis should be staged according to AJCC criteria.
- Where treatment may have an impact on fertility, patients should be referred to the appropriate reproductive medicine service before commencing treatment.

## Overview of management

As well as having care delivered or supervised by a specialist bone sarcoma MDT, patients should be allocated a key worker. Children, teenagers and young adults should also be discussed at the relevant children’s or TYA (young adult) MDT. This requires sufficient specialist staff to ensure age-appropriate care. A bone sarcoma MDT should be properly constituted, adhering to the requirements for core membership of the relevant specialties, and meeting minimum criteria for the number of patients treated each year; they should collect data on patients, tumours, treatment and outcomes as agreed nationally and participate in national audit.

Where possible and where trials are available, patients should be supported to participate in clinical trials. Lists of clinical trials in the National Institute for Health Research portfolio can be found on the UK Clinical Research Network Study Portfolio website [70] and the National Cancer Research Institute Clinical Trials website [71].

### Chemotherapy

Chemotherapy is part of standard treatment for osteosarcoma, Ewing sarcoma, undifferentiated pleomorphic sarcoma and spindle cell sarcoma. Treatment of chondrosarcoma remains predominantly surgical, although chemotherapy may have a role in dedifferentiated and mesenchymal subtypes.

Management usually comprises preoperative neoadjuvant systemic combination chemotherapy, local surgery and post-operative adjuvant chemotherapy [64]. While the main aim of neoadjuvant chemotherapy is to decrease the incidence of a subsequent distant relapse [72, 73], it may also help control the primary tumour.

### Surgery

Decisions about the optimal surgical procedure for the primary tumour (i.e. limb salvage or amputation) require MDT discussion, considering tumour size and involvement of anatomical structures, response to neoadjuvant therapies and patient preference. Surgical reconstruction may be influenced by patient and surgeon choice and should follow open discussion of the risks and benefits of available options and expected functional outcomes.

The aim of curative surgery is to resect the whole tumour with adequate margins. Where possible, wide *en*-*bloc* resection of the affected part of the bone and involved soft tissue should be performed. Close surgical margins may be marked with (MRI-inert) haemo-clips placed in the surgical field. In Ewing sarcoma, surgery should involve removal of all anatomical structures involved in the original prechemotherapy tumour volume where feasible. The specimen should be orientated to allow the pathologist to describe the anatomical location and thickness of surgical margins.

Surgical excision of local recurrence or metastatic disease requires discussion in a bone sarcoma MDT.

#### Requirements for the surgical report

The surgeon should describe the procedure performed and the tissues resected. The planned surgical margin should be identified, along with areas of concern where the resection was close to tumour or gross tumour was encountered. The type of reconstruction should be described as well as postoperative care, including expected rehabilitation. The use of prophylactic antibiotics and thromboprophylaxis (e.g. mechanical and/or chemical agents) should be clearly stated.

### Radiotherapy

Radiotherapy is frequently used in the definitive management of the primary tumour for Ewing sarcoma, but the relative radio-resistance of osteosarcoma and chondrosarcoma means it is only used as definitive treatment if there is no surgical option. Radiotherapy is not given routinely post-operatively, although it may be used in selected high-risk cases. However, radiotherapy has a palliative role in all tumour types.

Although considered exploratory, heavy particle therapy with protons or carbon ions, often in combination with photons, is increasingly used to treat unresectable primary bone sarcomas [74–76]. Excellent outcomes are reported for skull base chondrosarcomas or chordomas in which proton beam radiotherapy combined with surgery can achieve local control rates of approximately 70–90 % [77–79]. In unresectable or incompletely resectable osteosarcoma the five-year disease free survival (DFS) was 65 %, and the 5-year overall survival (OS) was 67 % [80]. High local control rates have also been achieved in sacral chordomas [81, 82].

At present there is no proton facility in the United Kingdom, but cases can be submitted to the UK Proton Panel to consider funding for treatment overseas. Referral guidelines can be found at the UK NHS England Commissioning website [83].

### Prevention and management of pathological fracture

Patients with an existing or impending pathological fracture associated with a suspected primary bone tumour should be managed with external splintage or immobilisation and appropriate pain control until a diagnosis is established by local imaging (MRI and/or CT) and biopsy. Internal fixation is contraindicated.

Although pathological fracture is an adverse prognostic factor for survival in osteosarcoma, and is likely to be associated with an increased risk of local recurrence [84], it does not preclude limb sparing surgery [85].

Fractures often heal during neoadjuvant chemotherapy and allow subsequent resection of the tumour and involved soft tissues. Amputation may still be indicated if tumours fail to show a radiological response and/or resection of the tumour and the contaminated area cannot safely leave a useful limb [86]. Adjuvant radiotherapy may decrease the risk of local recurrence in osteosarcoma and may have a role in other tumour types after pathological fracture [87].

### Thoracotomy

Pulmonary metastatectomy may be indicated in the presence of oligometastatic disease where the patient can be rendered disease free. Thoracotomy with manual exploration of both lungs is strongly recommended, even when imaging studies suggest unilateral disease. Thoracoscopic techniques are strongly discouraged, as they lack sensitivity and may be associated with an increased risk of intraoperative tumour dissemination [88].

### Key recommendations

- All patients with a confirmed diagnosis of bone sarcoma should have their care supervised by a bone sarcoma MDT and be allocated a key worker. Children, teenagers and young adults should also be discussed at the relevant children’s or TYA (young adult) MDT.
- Networks should ensure that they meet the needs of children and young people with cancer with sufficient specialist staff and care and facilities appropriate to the child or young person’s age.
- A bone sarcoma MDT should meet minimum criteria for the number of patients treated in each year and adhere to the requirements for core membership of the relevant specialties.
- All bone sarcoma MDTs should collect data on patients, tumours, treatment and outcomes as agreed nationally.
- Patients should undergo definitive resection of their sarcoma by a surgeon who is a core or extended member of a bone sarcoma MDT or by a surgeon with tumour site specific or age appropriate skills in consultation with the bone sarcoma MDT.
- When considering the local treatment of bone tumours, options for amputation or limb sparing surgery should be tailored to the needs of the patient.
- Chemotherapy and radiotherapy are important components of the treatment of some patients and should be carried out at designated centres by appropriate specialists as recommended by a bone sarcoma MDT.
- For pulmonary metastatectomy, open thoracotomy is recommended over endoscopic techniques.

## Specific treatment

### Osteosarcoma

Adverse prognostic factors for osteosarcoma include detectable metastases at presentation, axial or proximal extremity tumour site, large tumour volume, elevated ALP or LDH, older age, high body mass index (BMI) at diagnosis, poor histological response to preoperative chemotherapy or pathological fracture [62, 89–91]. There is some evidence that females may have better outcomes than males [91] and patients >18 years may have poorer outcomes than younger patients [92].

#### Localised disease

Curative treatment for high-grade osteosarcoma consists of surgery and chemotherapy [88, 93]. Compared with surgery alone, multimodal treatment of high-grade osteosarcoma increases survival from only 10–20 % to around 60 % [94, 95]. Whenever possible, patients with osteosarcoma should receive chemotherapy within a prospective trial. Chemotherapy is also recommended for older patients with osteosarcoma using adapted protocols [96].

Treatment commonly takes 6–9 months, comprising 10 weeks of neoadjuvant therapy, surgical resection and adjuvant chemotherapy.

Although neoadjuvant treatment is not proven to add survival benefit over postoperative chemotherapy alone, advantages include: rapid improvement in symptoms; early treatment of micrometastatic disease; facilitation of resection in responsive tumours; it allows time to manufacture customised endoprosthesis and provides prognostic information about histological response [42, 45, 97].

The most accepted regimen is induction therapy with MAP (high-dose methotrexate (HDMTX), doxorubicin and cisplatin). This is recommended in the UK for patients with potentially resectable tumours [98] and was chosen for the EURAMOS study^1^ (Table 3).

**Table 3:** Sarcoma advisory group guidelines for osteosarcoma

Category	1st line	2nd line	3rd line and other
Resectable <30 years	Doxorubicin, cisplatin methotrexate ± mifamurtide [101]	Ifosfamide and etoposide [102]	Gemcitabine and docetaxel [103] or oral etoposide
Other	Doxorubicin, cisplatin ± methotrexate [101]	Ifosfamide, etoposide ± methotrexate	Gemcitabine and docetaxel [103] or oral etoposide

Taken from: London and South East Sarcoma Network (LSESN) Guidelines [98]

If not tolerated, the regimen may be modified to AP alone for patients >40 years old. Impaired renal function can cause delayed clearance of methotrexate resulting in mucositis and nephrotoxicity and therefore close monitoring is required. Combination regimens without methotrexate can be effective in patients intolerant of HDMTX or where pharmacokinetic monitoring is not available [99].

The goal of surgery is to safely remove the whole tumour whilst preserving as much function as possible. Most patients with extremity tumours are candidates for limb salvage if adequate surgical margins can be achieved. Where possible, wide surgical margins should be achieved to reduce the risk of recurrence [100]. It is accepted that a good (>90 %) histological necrosis rate after chemotherapy may allow a closer margin of excision to be considered safe. In patients with a poor response to chemotherapy and ‘close’ margins there is insufficient evidence to advise as to whether amputation offers a better outcome even accepting the increased rate of local recurrence with limb salvage [86].

The benefit of adjuvant therapy compared with surgery alone was demonstrated many years ago [104] and long-term (>25 years) follow-up has shown that a statistically significant survival benefit is maintained [105]. Adjuvant therapy may involve the same regimen as the induction phase or may be modified, but the ideal combination regimen and the optimal treatment duration for certain clinical situations are yet to be defined [93, 106].

Immune modulation has been proposed as a possible treatment in bone sarcomas. The immune modulator liposomal muramyl tripeptide (mifamurtide) added to postoperative chemotherapy demonstrated a statistically significant advantage in overall survival and a trend in event-free survival in a large randomised trial [101] and has been approved in Europe for patients under 30 with completely resected localised osteosarcoma.

Interferon has also been investigated in in vitro and xenograft models [107] but its evaluation in the EURAMOS-1 trial showed no apparent advantage [108, 109].

Histological response to induction therapy has been accepted as a robust prognostic indicator [62, 91, 100, 110]. Imaging techniques to identify response preoperatively, such as FDG-PET [111] and dynamic (diffusion weighted) MRI [112] are under investigation.

Changing postoperative chemotherapy on the basis of response has not been shown to improve outcome, and is not recommended at present [42].

The use of haematopoietic growth factors to increase dose intensity has not consistently resulted in improved survival of osteosarcoma patients [95] but may limit morbidity associated with myelosuppression. Prophylactic antibiotics are now recommended for cancer patients at risk of neutropenic sepsis [113].

#### Central, parosteal and craniofacial osteosarcomas

Low-grade central and parosteal osteosarcoma are variants with lower malignant potential, for which treatment is surgical. Histological examination of the resected tumour may show high grade areas in which case treatment should be with chemotherapy as for conventional osteosarcoma.

The exact role of chemotherapy has not been defined for periosteal and jaw osteosarcoma but experience shows that standard chemotherapy can be given and should be considered for all patients at presentation as part of evaluation by an experienced MDT. Jaw and other craniofacial osteosarcomas present specific problems for management, especially to achieve local control, and must always be referred to a bone sarcoma MDT before surgery. ^18^FDG PET is more reliable than standard imaging in evaluating response to neoadjuvant chemotherapy in craniofacial bone sarcomas and may correlate better with outcome than histological response [114].

#### Metastatic disease

Patients presenting with metastatic osteosarcoma are a heterogeneous group and may be treated using the same regimens as for non-metastatic osteosarcomas, provided that surgical resection of all disease sites is deemed feasible [115]. Approximately 30 % of patients with primary metastatic osteosarcoma and over 40 % of those who achieve complete surgical remission become long-term survivors [93].

#### Recurrent disease

The prognosis for recurrent disease is poor, with long-term post-relapse survival of less than a third [93]. Early relapse and distant non-lung metastases are associated with a poorer prognosis [116].

Treatment for locally recurrent or metastatic osteosarcoma is primarily surgical, if possible. Pulmonary metastatectomy can lead to long term survival if all metastases can be completely removed [117]. More than a third of patients with a second surgical remission survive for over 5 years, and patients with multiple recurrences may be cured as long as recurrences are resectable: repeated thoracotomies are often warranted [118]. However, if pulmonary metastases are inoperable the disease is almost universally fatal.

The role of second-line chemotherapy for recurrent osteosarcoma is less well defined than that of surgery and there is no accepted standard regimen [93, 115]. The choice of agents may take into account the prior disease-free interval; suggested regimens are shown in Table 3 [98].

Second-line chemotherapy is associated with limited prolongation of survival in patients with inoperable metastases, but a positive benefit in operable disease was observed in one series [119–121]. Radiotherapy, including samarium, may palliate inoperable sites [4, 122].

Agents targeting the epidermal growth factor receptor are investigational in patients with unresectable disease failing first-line therapy [123].

#### Treatment evaluation

Assessment of response is usually only possible after several cycles of chemotherapy. Changes in the size and ossification of the tumour do not reliably reflect response to neoadjuvant chemotherapy. However, reduction in peritumoural oedema seen on MRI indicates a good treatment response [124]. A small study showed PET/CT is more accurate than MRI for following bone lesions [58, 125] but confirmation in larger prospective studies is needed.

### Key recommendations

- Treatment for osteosarcoma involves chemotherapy and surgery under the care of a specialist bone sarcoma MDT.
- Patients should be informed about relevant clinical trials and supported to enter them.
- First line standard treatment is MAP chemotherapy for patients under 40 years.
- Mifamurtide may be offered to patients without metastases after surgery.
- Treatment of the primary tumour should be surgical removal of the tumour with negative surgical margins where feasible.
- The adequacy of local clearance should be assessed by considering the response to chemotherapy and the surgical margin.
- Radiotherapy can be offered for local control where surgical removal is not possible.
- Where pulmonary metastases are present successful excision may prolong survival.
- The primary treatment of recurrent disease is surgical although there is a role for chemotherapy.

### Ewing sarcoma

Prognostic factors for Ewing sarcoma include axial location, tumour volume, raised serum LDH, and older age (>15 years). A poor histological response to preoperative chemotherapy and incomplete or no surgery for local therapy are further adverse prognostic factors [89, 126–128].

Ewing Sarcoma is a radiosensitive tumour. Radiotherapy may be used in combination with surgery, where there is a poor response to chemotherapy (radiological or histological), if there are concerns about surgical resection margins [129, 130] or if the anatomical site makes complete resection impossible. Radiotherapy may be given to the primary tumour site preoperatively, postoperatively or as definitive local therapy where surgery is not possible.

With surgery or radiotherapy alone, 5-year survival for Ewing sarcoma is <10 %. With treatment in current multimodality trials including chemotherapy, 5-year survival is between 60 and 70 % in localized and 20 to 40 % in metastatic disease [4].

#### Localised disease

All current trials employ 10 to 12 months of treatment comprising three to six cycles of neoadjuvant chemotherapy, followed by local therapy and a further six to ten cycles of chemotherapy usually given at 2 or 3 week intervals and based on current agreed national or international protocols. Agents considered most active include doxorubicin, cyclophosphamide, ifosfamide, vincristine, dactinomycin and etoposide [23, 89, 127, 130–139]. The LSEN guidelines [98] are summarized in Table 4. Virtually all active protocols are based on four to six drug combinations of these agents. Chemotherapy intensity is positively associated with outcome: superior outcome has been demonstrated with compressed 2 weekly chemotherapy [140]. High dose chemotherapy with blood stem cell transplantation is still investigational [141].

**Table 4:** Sarcoma advisory group guidelines—ewing sarcomas

Category	1st line	2nd line	3rd line and other
Localised or metastatic disease with lung or pleural metastases only	VIDE 6 cycles VAC or VAI × 8 cycles [142]	High dose ifosfamide [144] or Ifosfamide and etoposide or cyclophosphamide and topotecan [145] or irinotecan and temozolamide [146] ± High dose chemotherapy (busulphan/lmelphalan or treosulphan/melphalan) with peripheral blood stem cell rescue [147] N.B. Choice of 2nd line therapy will depend on patient/disease specific factors (e.g. if chemo being given with curative intent) and/or patient choice	Cyclophosphamide and topotecan [145] or Irinotecan and temozolamide [146] or gemcitabine and docetaxel [148, 149] or oral etoposide [150] or cisplatin and etoposide
Metastatic disease with bone or bone marrow involvement	VIDE × 6 cycles → VAC or VAI × 8 cycles [142, 143] or VDC/IE interval compressed regimen × 14 cycles [140]		

Taken from: London and South East Sarcoma Network (LSESN) Guidelines [98]

*V* vincristine; *I* ifosfamide; *D* doxorubicin; *E* etoposide; *A* actinomycin *D* (dactinomycin); *C* cyclophosphamide

Local treatment may comprise surgery or radiotherapy or both. Individual decisions about local therapy are frequently complex and should only be made by a bone sarcoma MDT in conjunction with the patient and their family if appropriate. It is also recommended that the MDT’s treatment plan is then discussed at the UK National Ewings MDT (ROH-tr.ewingsMDT@nhs.net) and entry into a clinical trial considered. At the time of writing (2015), the EE2012 trial is still open [151].

Complete surgery is regarded as the best treatment for local control but may not always be feasible. There is increasing recognition of the importance of treating all tissue initially involved by tumour, even if there has been a good response to chemotherapy. If this volume cannot confidently be removed surgically then radiotherapy should be used.

Indications for planned preoperative radiotherapy include poor response to induction chemotherapy, expected marginal resection, or if radiotherapy is anticipated to be required and the bone sarcoma MDT judges there is a technical advantage to preoperative radiotherapy.

Preoperative radiotherapy may also be useful in particular anatomical locations (e.g. pelvis, rib) when preoperative treatment allows the tumour volume to be defined more easily, or when treatment volumes will be smaller than in the post-operative setting. Radiotherapy alone should be considered if complete surgery is impossible or would be very disabling, (e.g. for sacral tumours crossing the midline) [152, 153]. If standard conformal radiotherapy will not achieve an adequate dose to the tumour, techniques such as IMRT (intensity modulated radiotherapy) may deliver a higher dose [154–156]. Insertion of pelvic spacers can displace bowel away from pelvic tumours, facilitating delivery of a higher dose and preventing long term bowel toxicity [157]. Proton beam radiotherapy may be considered when there is a dosimetric advantage over photon radiotherapy in achieving the optimal radiotherapy dose due to proximity to critical structures such as spinal cord, and for younger patients having curative treatment in order to reduce the risk of radiation-induced second malignancy. Applications for treatment are made via the UK Proton Panel [83]).

Toxicities leading to death have been observed in some patients who received high dose large volume radiotherapy following busulpan-melphalan high dose chemotherapy (BuMel HDT). BuMel HDT may therefore compromise the delivery of effective radiation doses to central axial sites. In patients with an indication for radiotherapy, the patient should not be offered BuMel HDT if there are critical organs such as gut, spinal cord, brain or significant volumes of lung in the fields, unless the technique used can limit the dose to critical organs.

Specific indications for post-operative radiotherapy include (Taken from: Euro-Ewing-2012 radiotherapy guidelines [158]):

- positive surgical margins with microscopic residual disease (R1 excision; <1 mm or tumour up to edge of resection specimen) if further surgery to achieve negative margins is not possible.
- positive surgical margins with macroscopic residual disease (R2 excision), if further surgery to achieve negative margins is not possible (this should be an unusual situation).
- if all tissues involved by the original pre-chemotherapy tumour volume have not been excised, even if the surgical margins are negative.
- if there is a poor histological response (≤90 % necrosis) to pre-operative chemotherapy, even if the surgical margins are negative.
- a displaced pathological fracture of bone at primary site (unless it is possible to excise all contaminated tissue).
- certain tumour sites where local control is judged to be more difficult to achieve e.g.:
  - Spine and paraspinal sites—in these sites excision is rarely complete, and is often intra-lesional.
  - Pelvis and sacrum—in these sites it is frequently difficult or impossible to be sure that the entire pre-chemotherapy tumour volume has been excised.
  - Rib tumours when presenting with a pleural effusion.

Reasons for deciding *against* radiotherapy may include:

- Concerns about impaired healing of the wound or biological reconstruction following surgery and radiotherapy.
- Concerns about morbidity of radiotherapy in young patients.
- Concerns about the increased risk of infection of a metallic prosthesis following radiotherapy.
- Concerns about the risk of a radiation-induced malignancy.

Definitive radiotherapy is advised only for inoperable lesions. Inoperability is determined during bone sarcoma MDT discussion. Inoperable tumours are those which cannot be resected completely, or are in anatomical sites where complete surgery would result in unacceptable morbidity or have a high risk of significant complications. Suggested radiotherapy doses are given in Table 5.

**Table 5:** Radiotherapy dose and fractionation for ewing sarcoma

Setting	Dosage
Pre-operative radiotherapy	The total dose for preoperative irradiation is 50.4 Gy in 28 fractions in a single phase to the PTV. If there are concerns about organ tolerance or wound healing, then this dose can be reduced to 45 Gy in 25 Gy fractions
Post-operative radiotherapy	The total dose for postoperative radiotherapy is 54 Gy in 30 fractions, delivered as 45 Gy in 25 fractions to PTV1, and 9 Gy in 5 fractions to PTV2
Definitive radiotherapy	The total dose for definitive radiotherapy is 54 Gy in 1.8 Gy fractions, delivered as a single phase. A boost of 5.4 Gy in 3 fractions may be considered if desired, keeping within standard normal tissue dose constraints

Fractionation: conventionally fractionated radiotherapy (once daily fractions, five 1.8 Gy fractions per week) is the preferred fractionation schedule. In very young children, fractionation using 1.6 Gy fractions may be considered

Taken from: Euro–Ewing-2012 radiotherapy guidelines [158]

#### Metastatic and recurrent disease

Around 26 % of patients with Ewing sarcoma have metastatic disease at presentation (10 % lung, 10 % bones/bone marrow, 6 % combinations or others) [159]. Bone metastases confer a poorer outcome than lung/pleural metastases (<21 % compared with 55 % 5-year relapse free survival) [143].

Patients with metastases at diagnosis are treated similarly to those with localised disease but have a poorer prognosis. Several non-randomised trials have evaluated more intensive, time compressed or high-dose chemotherapy approaches, followed by autologous stem cell rescue, demonstrating a possible advantage for patients under 14 years of age [143, 147]. Whole lung radiotherapy is indicated in patients with pulmonary disease, and may prolong survival. However, firm data are lacking and a systematic review failed to confirm a survival advantage [160]. Radiation doses are given in Table 6.

**Table 6:** Radiotherapy doses for whole lung radiotherapy

Whole lung radiotherapy	The dose for whole lung radiotherapy is 15 Gy in 10 fractions for patients <14 years, or 18 Gy in 12 fractions for patients ≥14 years. Dose may be specified to 100 % for an optimised plan, or to the mid plane dose (MPD) for simulated opposed fields. However, it should be noted that this will result in a dose of approximately 10 % higher in the lungs than that prescribed, and so optimisation of dosimetry is recommended if fields are simulated

Taken from: Euro–Ewing-2012 Radiotherapy Guidelines [158]

A recent review of stereotactic body radiotherapy (SBRT) for metastatic and recurrent Ewing sarcoma and osteosarcoma reported on 14 patients with 27 osseous or pulmonary lesions. Estimated local control at 2 years in the lesions treated with curative intent was 85 %. However, there was significant toxicity especially if concurrent chemotherapy and re-irradiation were given [161].

The role of surgical resection of residual metastases is less well defined. Patients with bone or bone marrow metastases and patients with recurrent disease still fare poorly, with 5-year survival rates of between 10 and 45 % [24, 162, 163].

Guidance on the management of small suspicious lung nodules is available in trial protocols [108, 131].

Patients relapsing more than 2 years after diagnosis and without bone marrow or multiple bone involvement have a better outcome than others [126, 162, 164, 165].

Doxorubicin therapy is usually not feasible after relapse because of previously administered cumulative doses. Chemotherapy regimens are not standardised and currently often comprise alkylating agents (cyclophosphamide, high-dose ifosfamide) in combination with topoisomerase inhibitors (etoposide, topotecan) or irinotecan with temozolomide [23, 133, 137, 138]. Given the poor outcomes after relapse, patients should be recruited to prospective clinical trials to investigate the role of second-line/experimental therapies wherever possible. At time of writing, an international trial, rEECur, is open to recruitment [151]. Radiotherapy may be helpful to palliate local symptoms.

#### Treatment evaluation

Change in the size of the soft tissue mass is easily evaluated on MRI, and is a reliable indicator of tumour response [166]. Dynamic MRI is not as reliable as in osteosarcoma, as remaining small tumour foci may not be detected. Sequential FDG PET evaluation and whole body MRI scanning is under evaluation [53, 112].

Disease progression during chemotherapy may mandate changes in treatment or earlier primary local control measures. A radiological increase in tumour size may be due to necrosis rather than tumour progression.

### Key recommendations

- For Ewing sarcoma, systemic treatment with chemotherapy is standard. All new cases of Ewing’s sarcoma of bone should be discussed at the National Ewing Multidisciplinary Team meeting.
- When treating the primary tumour with curative intent, all of the pre-chemotherapy volume should be treated with surgery, radiotherapy or both.
- If radiotherapy is indicated (e.g. the anatomical location of the tumour makes complete resection impossible or there has been an incomplete response to chemotherapy as identified radiologically), then preoperative radiotherapy may be advantageous.
- Patients with relapsed/progressive disease should be considered for clinical trials.

### Chondrosarcoma

Assessing the grade of chondrosarcomas is difficult and variation in opinion is common, even between experts [28]. The diagnosis of chondrosarcoma requires discussion in a bone sarcoma MDT. Surgery is the treatment of choice.

Low grade cartilage tumours may recur locally but are unlikely to metastasise. Biopsy-confirmed low grade central chondrosarcomas in extremity long bones can be managed by complete curettage with or without adjuvant measures (e.g. phenol, cement, cryotherapy) with a high chance of success. Low grade peripheral chondrosarcomas (arising from osteochondromas) should be completely surgically removed, aiming to excise the tumour with a covering of normal tissue.

Higher grade chondrosarcomas (including clear cell chondrosarcoma) and all chondrosarcomas of the pelvis or axial skeleton should be surgically excised with wide margins [29, 30].

Mesenchymal chondrosarcoma may be responsive to chemotherapy and some patients may be considered for adjuvant or neoadjuvant therapy [114]. There is uncertainty about the chemotherapy sensitivity of dedifferentiated chondrosarcoma but it can be treated like osteosarcoma, although survival is poorer [31]. Survival after a diagnosis of dedifferentiated chondrosarcoma remains dismal. Complete excision is recommended if feasible, but there is a very high risk of local recurrence following pathological fracture. If wide margins cannot be reliably achieved with limb salvage, then amputation may maximize the chances of local control but there remains a high risk that metastases will develop.

### Key recommendations

- Diagnosis of a chondrosarcoma requires discussion in a bone sarcoma MDT.
- Management of chondrosarcoma is surgical excision with wide margins for all but low grade central limb chondrosarcoma where curettage may be adequate.
- There are no data to support the routine use of chemotherapy.

### Undifferentiated pleomorphic sarcomas.

Treatment strategies mimic those of osteosarcoma, with age-adjusted chemotherapy and complete *en*-*bloc* resection including any soft tissue component if possible.

### Chordoma

Assessment in a specialist centre with expertise in managing chordomas is essential. To date, conventional therapy for chordoma has been complete surgical resection [167]. High dose radiotherapy using proton beams or carbon ions may be used post-operatively, and are promising alternatives to surgery for some patients, particularly those with high sacral tumours [74, 81, 82].

Surgical excision of tumours of the skull base or cervical spine should aim to remove as much tumour as possible, whilst preserving neurological function and therefore quality of life. R0 resection is rarely possible. Eight studies (summarized by Stacchiotti et al. [35]) have shown that surgery (R1 and R2 resections) followed by radiotherapy in selected patients produced 5-year estimated overall survival of 55–86 % in patients with chordoma of the skull base and/or cervical spine.

Metastases are rare but local recurrence is common and difficult to cure [168]. Treatment for local recurrence may include surgery and/or radiation therapy and/or systemic treatment (Table 7) [4, 35]. Molecular targeted agents may be effective [169, 170].

**Table 7:** Sarcoma advisory group guidelines—bone sarcomas

Sarcoma type	Category	1st line	2nd line	3rd line and other
Other high grade bone sarcomas including malignant fibrous histiocytoma, leiomyosarcoma, angiosarcoma, spindle cell sarcoma, dedifferentiated chondrosarcoma		Doxorubicin, cisplatin ± methotrexate [101]	Ifosfamide, etoposide ± methotrexate [102]	Gemcitabine and docetaxel [103]
Giant cell tumour	Locally advanced unresectable/metastatic	Denosumab [175]		
Chordoma	Locally advanced, unresectable or metastatic: non-dedifferentiated dedifferentiated	Imatinib [171] doxorubicin or doxorubicin and cisplatin [176]	Addition of sirolimus [170] or Sunitinib [177]	

Taken from London and South East Sarcoma Network (LSESN) guidelines [98]

### Other bone sarcomas

Prognosis and prognostic factors after a diagnosis of spindle cell sarcoma are similar to those of patients with osteosarcoma [41, 171, 172]. Treatment should be similar.

Adamantinoma is a malignant tumour occurring in the tibia. Most are low grade but higher grade areas in the primary tumour may require systemic therapy. Complete excision is the treatment of choice.

### Giant cell tumours of bone

Giant cell tumours of bone require highly specialised treatment and all patients should be referred to a specialist bone sarcoma MDT for diagnosis and to coordinate treatment. Curettage alone is associated with a high risk of local recurrence (up to 50 %). Although there is no randomized controlled trial evidence, numerous case series suggest improved local control if adjuvants such as high speed burring and cement are used.

Denosumab is a novel RankL inhibitor which has been shown in clinical trials to suppress the formation and activity of osteoclasts. It is licensed for use by the European medicines agency from 2014. Denosumab is indicated in inoperable cases or those where the morbidity of surgery would be excessive. Denosumab is given as a monthly subcutaneous injection after three loading doses at weekly intervals. All patients require daily calcium and vitamin D supplements and females must avoid pregnancy. Significant side effects include hypocalcaemia, osteonecrosis of the jaw and atypical fractures [173, 174].

Emerging evidence suggests that whilst initial control is excellent (96 %), later recurrences can arise and most tumours recur if the drug is stopped (after around 9 months). Hence in inoperable cases life-long treatment may be required. The consequences of this, particularly in younger patients, are not known.

Using denosumab to reduce the size of a giant cell tumour prior to surgery may be advantageous but surgery should incorporate the extent of the original tumour to avoid recurrence. While clear guidance on the optimal duration of pre-operative treatment has yet to emerge, prolonged exposure to denosumab may make subsequent curettage more difficult. Treatment for up to 6 months before surgery is a reasonable pragmatic approach.

## Follow-up

Follow-up after treatment aims to detect local recurrence, to detect metastatic disease for which treatment might be beneficial, to manage the long term toxicity of chemotherapy and radiotherapy and to look for long term complications of surgical treatment [4]. Local recurrences are often first detected by patients and therefore they should be given information about what to do if local recurrence is suspected.

The clinical follow-up of patients treated for high-grade tumours should include physical examination of the primary tumour site, and assessment of the functional outcome and possible complications of any reconstruction. Local and chest imaging should be included. Evidence for the optimum frequency of follow-up and the best imaging investigations is lacking although a recently reported randomised controlled trial showed no benefit of greater frequency of follow-up with regular cross sectional imaging over standard follow-up [178]. However, current protocols recommend follow-up at intervals of 2–4 months for the first 3 years after completion of therapy, every 6 months for year 4 and 5 and annually thereafter [24, 93].

For low grade bone sarcomas, the frequency of follow-up visits can be reduced to 4–6 monthly for 2 years and then annually. Late metastases as well as local recurrences and failure of reconstructions may occur more than 10 years after diagnosis in all tumours and there is no universally accepted stopping point for follow-up [4].

It is important to evaluate the long-term toxicity effect of chemotherapy and radiotherapy as well as immediate chemotherapy-related complications [64]. Monitoring for late effects should be undertaken, depending on the treatment given and in conjunction with late effect services when available [127, 179, 180].

Secondary cancers may arise in survivors of bone sarcomas, either related to or independent of irradiation. Secondary leukaemia, particularly acute myeloid leukaemia may rarely be observed following chemotherapy as early as 2–5 years after treatment [181, 182].

### Key recommendations

- Standard follow-up for all sarcoma cases is currently chest X-ray and clinical review. The role of regular cross sectional imaging remains uncertain.
- At the end of treatment patients should receive information about the risk of local and systemic recurrence.
- Patients should have access to services for the late effects of treatment including chemotherapy, radiotherapy, surgery and psychsocial support.

## Footnotes

1 EURAMOS is a collaboration of the Children’s Oncology Group (US) the Co-operative Osteosarcoma Study Group (European) the European Osteosarcoma Intergroup and the Scandinavian Sarcoma Group. Collectively these groups represent the largest ever clinical experience in osteosarcoma.
